# Optimization of Urban Shelter Locations Using Bi-Level Multi-Objective Location-Allocation Model

**DOI:** 10.3390/ijerph19074401

**Published:** 2022-04-06

**Authors:** Lei He, Ziang Xie

**Affiliations:** Key Laboratory of Ecology and Energy-Saving Study of Dense Habitat (Ministry of Education), College of Architecture and Urban Planning, Tongji University, Shanghai 200092, China; leih@tongji.edu.cn

**Keywords:** location, shelter, site selection, disaster relief, optimization, bi-level programming

## Abstract

Recently, global natural disasters have occurred frequently and caused serious damage. As an important urban space resource and public service facility, the reasonable planning and layout optimization of shelters is very important to reduce the disaster loss and improve the sustainable development of cities. Based on the review of location theory and models for shelter site selection, this study constructs a bi-level multi-objective location-allocation model, an accessibility, economy, and efficiency (AEE) model, based on sequential decision logic to maximize the economic sustainability and social utility. The model comprehensively considers factors such as the level of decision-making, the utilization efficiency, and capacity constraints of shelters. The gravity model is introduced to simulate the decision-making behavior of evacuees. A calculation example and its solution prove the high practicability and operability of the AEE model in an actual shelter site selection and construction investment, which can achieve the global optimization of evacuation time and the maximization of the use efficiency of the shelters under the financial constraints. It provides a scientific and effective decision-making method for the multi-objective location optimization problem of shelters.

## 1. Introduction

Global climate change poses new challenges to the sustainable development of cities [1,2]. According to the report *“The Human Cost of Disasters 2000–2019”* issued by the UN Office for Disaster Risk Reduction (UNDRR, Geneva, Switzerland), there has been a sharp rise in climate-related disasters from 3656 climate-related events (1980–1999) to 6681 climate-related disasters in the period 2000–2019, which affected 3.9 billion people [3]. The impacts of climate change are being felt clearly in the increased frequency of extreme weather events and disasters. In this context, how to improve a city’s ability to respond to extreme disasters and reduce losses and casualties is a primary concern of governments [4].

As an important urban disaster prevention space and public service facility, the shelter is a resettlement measure for disaster victims in response to sudden incidents [5,6]. It is also a safe place for people in modern cities to escape the worst effects of earthquakes, floods, fires, explosions, and other major natural or accident disasters. After experiencing disasters such as the 2004 Indian Ocean Tsunami, the 2005 Hurricane Katrina, the 2008 Wenchuan earthquake, and the 2011 Tōhoku earthquake and tsunami, countries represented by Japan, the United States, and China have paid more attention to the scientific and rational planning of shelters, and incorporate it into urban planning and emergency system construction as an important content [7].

A reasonable site selection and construction scale can greatly improve the efficiency of emergency resettlements and the ability of cities to respond to emergencies. Otherwise, it may not only result in shortage or overutilization of shelters, but also may lead to economic unsustainability due to excessive planning and construction. After the 2008 Wenchuan earthquake, Mianyang Jiuzhou gymnasium, as an emergency shelter, received about 100,000 evacuees in a month, far exceeding the capacity of 6050 people, which caused great difficulties to the shelter life and emergency management [8,9]. In contrast, Shanghai plans to build 315 shelters by 2020 [10]. However, due to the large-scale government investment and high daily operation and maintenance costs, the actual construction is far from reaching the set goal and the resources between regions are unbalanced [11]. Therefore, research on the reasonable location and allocation of shelters has received much attention.

The shelter location problem essentially belongs to the public facility location problem. It refers to the selection of shelters from alternative sites (such as schools, stadiums, parks, public green spaces, and city squares) to meet the demands of the determined evacuation sites (such as residential areas, business areas, and factories). According to the disaster relief function, facility configuration, effective capacity, service area, and residence time, shelters can be divided into emergency, resident, and central shelters. Because emergency shelters undertake a temporary shelter function, their planning and location are relatively flexible and do not need special investment. Therefore, the research on the shelter location in this study mainly focuses on resident and central shelters. How to balance the demand and supply of shelters by considering the fairness, accessibility, and economics of urban investment? How to plan shelters based on humanism and the individual’s evacuation behavior? These are the main problems faced by the theory and practice of site selection for shelters [12,13].

Based on a systematic review of the location theory of shelter sites, this paper deduces that the decision-making level should be considered in the shelter site selection, and there is a certain relationship between superiors and subordinates. When choosing and building shelters from alternative sites, government decision-makers consider that the selected shelters have good suitability and low investment cost, so as to meet the shelter needs of all people under the limitation of evacuation distance. The evacuees choose the nearest shelter among the shelters determined by the government decision-makers. In view of the logic of decision-making, this paper constructs an accessibility, economy, and efficiency (AEE) bi-level optimization location model from the perspective of decision-makers and evacuees. The research conclusions can be used for the study of site selection planning and construction investment for urban shelters and can provide a scientific decision-making basis for shelter planning and evacuation strategies under limited finances. 

The remainder of this paper is organized as follows. Section 2 reviews the theory and methods of shelter location. Section 3 builds the AEE site optimization mathematical model. In Section 4, the practical application of the model is introduced, and the simulated annealing algorithm (SAA) is used to solve the model. Finally, Section 5 discusses the main contributions and conclusions.

## 2. Review of Location Theory and Optimization Model for Shelters

Shelter is an urban public facility that provides emergency evacuation services. The emergency facilities location problem was first proposed by Toregas in the 1970s [7,12,13,14,15,16]. However, the study of urban public facility locations has a history of hundreds of years [17]. Therefore, referring to the four-stage and the five-stage public facility location theory [18,19], this paper divides the shelter location theoretical research into three development stages. The authors review the location theory and optimization model and summarize the location optimization method, optimization objectives, and constraints.

### 2.1. Initial Period: Traditional Location Theory to L-A Model

Location theory has experienced three stages: classical, modern, and contemporary location theories [13,16,17,18,19]. From the classical location theory to the modern location theory, it has mainly focused on agriculture, industry, and commerce with the goal of minimizing costs or maximizing profits. The contemporary location theory that developed in the 1950s was no longer limited to cost and profit and began to focus on social benefits, providing a theoretical foundation for the study of the layout of public facilities [20]. Contrary to the previous industrial layout, the service objects and location selection of public facilities are determined by the government, and the public will all benefit fairly. These non-profit and government investment characteristics establish that the public facility location theory is different from the traditional location theory.

In the 1960s, Teitz first proposed the public facility location theory—fair allocation and maximum welfare of public facilities—which argued that the optimal layout of urban public facilities should consider fairness and efficiency [18]. In 1963, Cooper extended Weber’s industrial location theory to multiple location models in the field of public facilities and creatively put forward the location–allocation (L-A) model of public facilities [21]. The L-A model refers to the optimization of an objective function with certain constraints by quantifying the location principle. Several optimal sites are selected from a number of candidate sites, and the service areas of the facilities are scientifically and reasonably divided according to the capacity, accessibility, and evacuees’ selection. The essence of the L-A model is to solve the spatial relationship between supply and demand by optimizing facility location and allocation.

### 2.2. Quantitative Period: Construction and Development of the L-A Model

Fairness, efficiency, and cost are the three core issues of the L-A model. Focusing on a series of quantifiable indexes for evaluating fairness, efficiency, and cost, such as facility distance, accessibility, and the number of facilities, scholars used the operational research method to expand the Teitz public facility location model and develop the L-A model. In 1964, Hakimi proposed the P-median model (PMM) and solved the optimal location by linear programming, which began the quantitative research on the L-A model. Among these, the PMM [22,23,24,25,26,27], P-center model (PCM) [22,28], set covering location model (SCLM) [29,30,31,32], and maximum covering location model (MCLM) [33,34,35,36] are the four most widely used classical location models. The above classical models are single-objective determinate location models, with all parameters, such as service population, location, facility capacity, and construction and transportation costs, fixed in a certain period of time. From the perspective of shelter location, the optimization objectives, characteristics, advantages, and disadvantages of the four models are summarized, as shown in Table 1.

With the expansion of urban areas, the number of shelters and the complexity of evacuation road systems are increasing. After the 1980s, the Geographic Information System (GIS) was gradually applied to optimize site selection [37] to assist in analyzing the influence of complex spatial attribution factors on the location of shelters [38,39,40]. Although the combination of GIS technology and the L-A model does not improve the construction of the model, GIS’ powerful spatial analysis function promotes the realization of the model’s analysis and optimization methods.

### 2.3. Multiple Period: Complex and Diversified Location Research

The classical location model is a single-objective model, and its application in shelters is mainly aimed at the minimum total evacuation distance [25,41,42], the maximum service efficiency [34,35,43], or the minimum construction cost [29,30]. However, the actual shelter location problem is not affected by a single objective and constraint, and it should essentially be a multi-objective programming problem. Therefore, the multi-objective L-A model with a comprehensive consideration of multiple factors was developed. 

With the rise of multi-objective and multi-level public facility location research, the multiple criteria decision making (MCDM) method combined with qualitative and quantitative approaches has emerged as one of the main methods used in current shelter site selections. The Delphi method combined with fuzzy AHP [40,44,45,46,47], DEMATEL [48], and other methods are commonly used in MCDM to quantitatively evaluate the priority of influencing factors of shelters and score candidate sites. The principles generally focus on the safety, accessibility, capacity, connectivity, and economy of shelters. However, the deficiencies of MCDM are that the evaluation mainly determines the weight based on expert experience, does not consider the actual evacuation behavior and preference of evacuees, and is not applicable to the situation of uncertain quantity and site location.

After the initial achievements of shelter construction in most developing countries, research on shelter location turned to the reflection of “humanism” and began to re-examine the game relationship between government decision-making and the actual needs of evacuees. In 2005, Kongsomsaksakul et al. first proposed that the shelter location is a Starkberg game, which is a bi-level program. The leader (government) determines the location and number of shelters to minimize total evacuation time in the upper level, while the followers (evacuees) choose the shortest evacuation route in the lower level. Ng et al. [49], based on the research of Kongsomsaksakul et al. [24], optimized the allocation of shelters in the upper level and considered fairness as much as possible. Boonmee et al. [50] proposed a stochastic linear mixed-integer programming model based on the concept of bi-level programming. The lower level model considers the behavior of evacuees, shelter capacity, and uncertainty of the flooded area. In the current bi-level model study, the lower level mainly considers the shortest evacuation distance and gives less consideration to the individual preferences and satisfaction of the evacuees.

### 2.4. Summary and Evaluation

**Figure 1:**
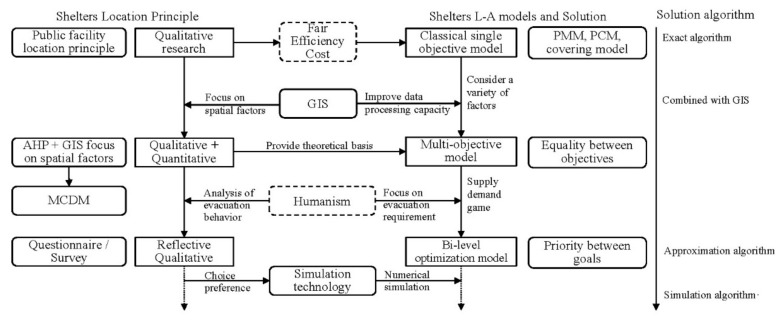
Development of shelter location research.

Throughout the history of shelter location and model development, the main objective of urban shelter site selection has been to achieve an “equalization of public services” within a limited budget. Scholars have always focused on this basic principle to strike a balance between fairness, efficiency, and cost (Figure 1, Table 2). The existing research can be divided into two categories. The first approach is to construct an L-A model and explore the model-solving algorithm by using operational research theory, which can accurately solve the problem of facilities’ location. However, solving the model is complex, and approximate intelligent algorithms such as genetic, iterative clustering, ant colony, and SAAs are often used. The second approach is to pay attention to the location principle and to determine the priority of shelter locations by using the MCDM that combines qualitative and quantitative methods. The conclusion can provide a theoretical basis for the optimization objective of the L-A model and location decision when the candidate sites are determined. Each of the above model methods has its own advantages, disadvantages, and application conditions. In contrast, the bi-level multi-objective L-A model is more suitable for the actual situation. The upper-level model considers the economy and fairness from the perspective of the government, while the lower-level model considers the accessibility from the perspective of the evacuees. However, at present, only the influence of the evacuation distance on the evacuees is considered.

There are three main objectives for the location of urban shelters in the above literature: ① The shelters cover all evacuation demand sites, and all evacuees can reach the shelter fairly; ② Minimize the total distances from the evacuation demand site to the shelter to achieve the maximum evacuation efficiency; ③ Minimize the number of shelters and reduce the investment. In addition, the constraints of the models include: ① Each evacuation demand site is allocated with a corresponding shelter; ② The time (distance) from the evacuation demand site to the shelter should be within certain limits; ③ Each shelter has a certain population capacity constraint; ④ Each shelter has a maximum service radius.

In summary, the existing studies on the shelter location model give insufficient consideration to the evacuees’ behavior and comprehensive economy. The application conditions limit the practical application value of the location model. This paper aims to construct a shelter location model to maximize the comprehensive utility. From the perspective of game theory, the government decision-making, evacuation behavior, and utilization and capacity constraints of shelters are considered. The bi-level multi-objective programming is used to minimize the cost of facility allocation and maximize the facility utilization rate and service population.

## 3. AEE Location Optimization Model

### 3.1. Model Concept

According to the existing research, this paper proposes five basic objectives of the shelter layout: safety, fairness, accessibility, economy, and efficiency.

(1) Safety is a basic prerequisite in built-up urban areas. Alternative sites for shelters such as schools, stadiums, parks, and green spaces meet the requirements of site security. Therefore, safety is considered to be a satisfied principle and not considered separately in the following study.
(2) Fairness means that all evacuees have shelters that meet the evacuation time constraints.
(3) Accessibility refers to the time to reach the shelter to meet the maximum evacuation time constraint. The accessibility of public facilities usually refers to the convenience of people with corresponding needs to reach the target facilities from a given location through some means of transportation. The fairness of the spatial layout of shelters is often reflected by the difference in public accessibility, which is a quantifiable index of fairness. Therefore, accessibility is used to characterize fairness in the following study.
(4) The economy of shelters is not exactly equivalent to the minimum number of shelters. In this study, the investment in shelters is a function of the number, scale, and unit construction cost of the shelters.
(5) Efficiency is quantified by the shortest total evacuation time.

From the perspective of economic and social utility, the above five objectives are conflicting and difficult to meet at the same time. Therefore, based on the three constraints of service capacity, evacuation time, and all people’s access to shelters, the model adopts sequential decision-making, with fairness first, total investment minimum, and overall evacuation efficiency optimal, so as to build a bi-level multi-objective optimization model called the AEE model.

(1) The upper-level model achieves the minimum investment: Construct an SCLM to meet the premise of covering all evacuation demand areas and obtain the number, scale, and location of shelters under the minimum investment.
(2) The lower level model achieves the shortest comprehensive evacuation time: Construct the PMM, optimize the evacuation route to improve the evacuation efficiency in the shelters determined by the upper layer, and try to meet the minimum evacuation route for all people.

### 3.2. AEE Mathematical Formulations

The upper-level model is given by

(1) $$\min\sum_{k=1}^{K}\sum_{i=1}^{3}m_{ik}y_{kj}$$

(2) $$\sum_{k=1}^{K}y_{kj}\;=\;1\;\forall j$$

(3) $$\sum_{k=1}^{K}t_{kj}y_{kj}\;\leq \;T_{\max}\forall j$$

(4) $$\sum_{j=1}^{J}h_{j}\cdot y_{kj}\;\leq \;z_{k}\;\forall k$$

(5) $$\gamma_{kj}\;=\;\alpha \frac{z_{k}\;\times \;hj}{t_{kj}^{2}}$$

(6) $$m_{ik}\;=\;a_{i}\;\times \;z_{k}$$

(7) $$x_{k}\;\in \;[0,\;1],\;y_{kj}\;\in \;[0,\;1]$$

In the above formulas, Equation (1) is the objective function, indicating that the shelter requires the minimum investment. Constraint (2) ensures that all evacuation demand sites are met, and any one of the evacuation demand sites is only allocated to one shelter. To facilitate management, the evacuees at an evacuation demand site are not split. One demand site corresponds to the same shelter, but one shelter can serve several demand sites. This is convenient for advance evacuation planning, making the management of the demand sites and shelters more efficient. Constraint (3) ensures that the evacuation time is within the maximum allowable time. Constraint (4) ensures that the total number of evacuees in each shelter does not exceed the maximum capacity. Equation (5) defines the relationship among the attractiveness, evacuation distance, evacuation population, and scale of the shelter based on the gravity model, where α is the adjustment coefficient and takes a value between 0 and 1; Equation (6) gives the construction cost of the shelter which corresponds to a unique level. According to the China national standards *Code for design of disasters mitigation emergency congregate shelter (GB 51143-2015)*: when 0.2 ≤ Sk ≤ 1 (ha), i = 1 is a resident short-term shelter; when 1 ≤ Sk ≤ 15 (ha), i = 2 is a resident long-term shelter; and when Sk ≥ 15 (ha), i = 3 is a central shelter. Equation (7) gives the decision variable restrictions.

Under the constraint condition, the upper model obtains the number of shelters *P*, the location of the shelter $x_{k}$, the shelter level *i*, the total investment *M*, and the initial evacuation route $y^{0}{}_{kj}$. Based on the identified shelter site, the allocation of evacuation demand sites is optimized and the minimum evacuation route $y_{kj}$ is obtained. 

The lower level model is given by

(8) $$\min\sum_{j=1}^{J}h_{j}\sum_{k=1}^{K}t_{kj}y_{kj}$$

(9) $$\sum_{k=1}^{K}x_{k}\;\leq \;P$$

(10) $$\sum m_{ik}\;\leq \;M$$

The objective Function (8) represents the shortest total time for all evacuees to take shelter. Constraint (9) indicates that the maximum number of shelters is *P* (obtained from the upper level). Equation (10) indicates that the total investment is less than the upper cost limit *M* (obtained from the upper level).

### 3.3. Model Parameters and Variables

According to the above-mentioned preset location rules, the relevant parameters and decision variables are determined as follows:

(1) Parameters

K = {k|k = 1,2,3,…,n} Set of alternative shelters; k ∈ K

J = {j|j = 1, 2, 3,…,n} Set of evacuation demand sites; j ∈ J

I = {i|i = 1, 2, 3} Set of shelters levels, *Code for design of disaster mitigation emergency congregate shelter GB 51143-2015* divides the refuge site into three levels. Each shelter level corresponds to different per-capita construction costs according to its area and corresponding facility allocation code; i ∈ I



$h_{j}$

Population of evacuation demand site *j*

$z_{k}$

Maximum evacuation capacity of shelter *k*

$s_{k}$

Effective evacuation area of shelter *k*

$a_{i}$

Per capita construction cost of shelter level *i* (constant) 

$t_{kj}$

Travel time from evacuation demand site *j* to shelter *k*

$\gamma_{kj}$

Attractiveness to evacuees from evacuation demand site *j* to shelter *k*

It is directly proportional to the population of evacuation demand sites and the construction scale of shelters and inversely proportional to the square of the shortest walking time between the evacuation demand sites and shelters.

$T_{max}$ Maximum allowable time for evacuees from the evacuation demand site to the shelter, which is equal to the maximum coverage distance of the shelter divided by the average walking speed of evacuees. Generally, the maximum allowable evacuation time for resident shelters is 10–15 min.

$m_{ik}$ Construction cost of the *i*-level shelter *k*, which is directly proportional to $z_{k}$ and $a_{i}$.

(2) Decision variables

$$\begin{array}{c} x_{k}=\left\{\begin{array}{l} 1,\;\mathrm{Shelter}\;k\;\mathrm{is}\;\mathrm{selected}\;\\ 0,\;\mathrm{Shelter}\;k\;\mathrm{is}\;\mathrm{not}\;\mathrm{selected}\; \end{array}\right.\\ y_{kj}=\left\{\begin{array}{l} 1,\;\mathrm{Evacuees}\;\mathrm{at}\;\mathrm{evacuation}\;\mathrm{demand}\;\mathrm{site}\;j\;\mathrm{are}\;\mathrm{allocated}\;\mathrm{to}\;\mathrm{shelter}\;k\;\\ 0,\;\mathrm{Otherwise}\; \end{array}\right. \end{array}$$

(3) Notes:

① The basis of the model application is to identify and generate the location matrix of all evacuation demand sites and shelters in the planning area. The land-use types of evacuation demand sites include residential areas. The land-use types of alternative shelters include parks, green spaces, squares, schools, rescue stations, playgrounds, stadiums, and social hotels. 

② According to the network topology, the network routes from all evacuation demand sites to all shelters are calculated, and the evacuation route and time matrices are generated.

③ $h_{j}$ is estimated according to the characteristics of the disaster situation and personnel composition at evacuation demand site *j*. 

④ $z_{k}$ is equal to $s_{k}$ divided by the per capita net sheltering area.

## 4. Calculation Example Using the AEE Model

### 4.1. Basic Situation of the Example

There are ten relatively concentrated residential areas (expressed as h_1_–h_10_) in a large urban development zone in China, with a total of 9400 people to be evacuated. The numbers of evacuees in each residential area are 1000, 1200, 1600, 2000, 400, 600, 200, 300, 1400, and 700. There are eight alternative shelter sites in the area (each land area is expressed as S_1_–S_8_), including one district-level park: S_6_ = 33,000 m^2^; two community-level parks: S_4_ = 6600 m^2^ and S_5_ = 13,000 m^2^; and five schools: S_1_ = 3500 m^2^, S_2_ = 3300 m^2^, S_3_ = 4000 m^2^, S_7_ = 4300 m^2^, and S_8_ = 5000 m^2^. The time from the residential area to each alternative shelter site is shown in Equation (14). The government wants to meet the evacuation demands of 9400 people in the region with the least amount of investment. Requirements: ① All people have access to shelters; ② The scale, level, and facilities of the shelters meet the demands of the evacuees; ③ The number of shelters is appropriate, and the total investment cost is a minimum; ④ The utilization rate of shelters is a maximum, and each investment can be used to the best of its ability.

**Figure 2:**
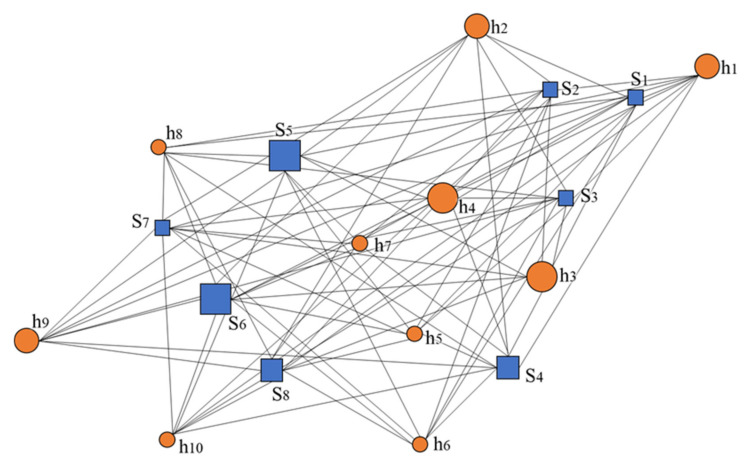
Schematic diagram of the L-A model.

The calculation example can be abstractly expressed as a schematic diagram, as shown in Figure 2. The squares represent alternative shelter sites, the circles represent residential areas, and the size of the graph is related to the actual area. The straight line represents the distance from the residential area to the alternative shelter, where the distance represents the shortest path distance based on the road network in practice.

### 4.2. Input Parameters

① The ten known residential areas with evacuation demands are expressed as |*J*| = 10, and eight alternative shelters are expressed as |*K*| = 8; the number of evacuees in each residential area is expressed as Equation (11).

② The effective and safe shelter area for the candidate sites is expressed as Equation (12), which is obtained by multiplying the land area of the candidate sites by the reduction factor (in this example, the reduction factor is 0.6).

③ The maximum population capacity of the candidate shelter sites is expressed as (13), which is obtained from Equation (12) in combination with the code. According to the *Code for design of disasters mitigation emergency congregate shelter GB 51143-2015*, the per capita net sheltering areas are short-term resident shelter 2 m^2^/per; long-term resident shelter 3 m^2^/per, and the central resident shelter is 4.5 m^2^/per.

④ The time matrix between the evacuation demand site and the shelter is shown as Equation (14), calculated from the actual shortest path based on the road network and the average pedestrian evacuation speed of 3 km/h.

⑤ The *γ_kj_* matrix is calculated according to Equation (5) (*α* is 1) and normalized as shown in Equation (15) (dimensionless); if *T_max_* ≤ 15 min, the *γ_kj_* corresponding to the route with an evacuation time greater than 15 min should be 0. When the number of evacuees in the residential area exceeds the maximum capacity of the candidate shelter sites, the corresponding *γ_kj_* shall be 0.

⑥ Assume that the per capita construction cost of the short-term resident shelter is 5000 yuan, the long-term resident shelter is 10,000 yuan, and the central shelter is 20,000 yuan. Then, *a*_1_ = 5000, *a*_2_ = 10,000, and *a*_3_ = 20,000, and *m_ik_* is given as Equation (16).

(11) $$h_{j}\;=\;\left[1000,1200,1600,2000,400,600,200,300,1400,700\right]$$

(12) $$S_{k}\;=\;{\left[2100,2000,2400,4000,8000,20000,2600,3000\right]}^{T}$$

(13) $$z_{k}\;=\;{\left[1050,1000,1200,2000,4000,6666,1300,1500\right]}^{T}$$

(14) $$t_{kj}\;=\;\left[\begin{array}{cccccccccc} 6 & 8 & 10 & 10 & 14 & 15 & 12 & 18 & 19 & 20\\ 10 & 6 & 10 & 8 & 15 & 17 & 12 & 16 & 20 & 21\\ 12 & 8 & 5 & 6 & 8 & 10 & 8 & 12 & 15 & 18\\ 15 & 12 & 5 & 8 & 5 & 7 & 8 & 10 & 13 & 15\\ 15 & 12 & 10 & 8 & 12 & 15 & 5 & 6 & 10 & 12\\ 20 & 18 & 16 & 15 & 13 & 15 & 10 & 8 & 10 & 5\\ 20 & 17 & 16 & 15 & 8 & 10 & 12 & 6 & 8 & 8\\ 20 & 16 & 13 & 15 & 6 & 5 & 9 & 10 & 8 & 11 \end{array}\right]$$

(15) $$\gamma_{kj}\;=\;\left[\begin{array}{cccccccccc} 0.83 & 0.00 & 0.00 & 0.00 & 0.06 & 0.08 & 0.04 & 0.00 & 0.00 & 0.00\\ 0.66 & 0.00 & 0.00 & 0.00 & 0.12 & 0.14 & 0.09 & 0.00 & 0.00 & 0.00\\ 0.16 & 0.43 & 0.00 & 0.00 & 0.14 & 0.14 & 0.07 & 0.05 & 0.00 & 0.00\\ 0.03 & 0.05 & 0.42 & 0.20 & 0.10 & 0.08 & 0.02 & 0.02 & 0.05 & 0.02\\ 0.04 & 0.08 & 0.16 & 0.31 & 0.03 & 0.03 & 0.08 & 0.08 & 0.14 & 0.05\\ 0.00 & 0.00 & 0.00 & 0.14 & 0.04 & 0.04 & 0.03 & 0.07 & 0.22 & 0.45\\ 0.00 & 0.00 & 0.00 & 0.00 & 0.19 & 0.18 & 0.04 & 0.25 & 0.00 & 0.33\\ 0.00 & 0.00 & 0.00 & 0.00 & 0.16 & 0.35 & 0.04 & 0.04 & 0.32 & 0.08 \end{array}\right]$$

(16) $$m_{ik}\;=\;\left[800a_{1},1000a_{1},1200a_{1},2000a_{1},4000a_{1},6666a_{2},1000a_{1},800a_{1}\right]$$

### 4.3. Example Solution

This problem belongs to the NP-hard problems, which is a problem for which we cannot prove that a polynomial-time solution exists. The SAA is used to obtain the optimal solution. The methods are as follows:

① First, randomly select n candidate sites as the initial solution and substitute the objective function to obtain the total input *M*_0_.

② Select *k* randomly from the remaining (|*K*|−*n*) candidate shelter sites, replace the random *j* in *n*, and substitute the objective function to obtain the total input *M*_1_.

③ Cycle the calculations until the obtained objective function *M* is the minimum value and does not change, and the corresponding shelter is the optimal solution of the upper-level model. 

④ The SAA is used to solve the lower-level model, and the evacuation demand sites are randomly exchanged to calculate the minimum evacuation time. When the objective function is minimized and stable, the optimal solution of the lower-level model is obtained.

The following results are obtained:

①$\min\sum_{k=1}^{K}\sum_{i=1}^{3}m_{ik}y_{kj}$ = 4750 means the minimum investment cost of the shelter is 47.5 million yuan.

② *X_k_* = [0,1,1,1,1,0,1,0]^T^, that is, the evacuation sites *k* = 2,3,4,5,7 are selected as the final shelters; the effective and safe area for each shelter is 2000 m^2^, 2400 m^2^, 4000 m^2^, 8000 m^2^, and 2600 m^2^; the shelter level is a short-term resident shelter, and the population capacities are 1000, 1200, 2000, 4000, and 1300.

③ Solving $y_{kj}$:

(17) $$y_{kj}\;=\;\left[\begin{array}{cccccccccc} 0 & 0 & 0 & 0 & 0 & 0 & 0 & 0 & 0 & 0\\ 1 & 0 & 0 & 0 & 0 & 0 & 0 & 0 & 0 & 0\\ 0 & 1 & 0 & 0 & 0 & 0 & 0 & 0 & 0 & 0\\ 0 & 0 & 1 & 0 & 1 & 0 & 0 & 0 & 0 & 0\\ 0 & 0 & 0 & 1 & 0 & 0 & 1 & 1 & 1 & 0\\ 0 & 0 & 0 & 0 & 0 & 0 & 0 & 0 & 0 & 0\\ 0 & 0 & 0 & 0 & 0 & 1 & 0 & 0 & 0 & 1\\ 0 & 0 & 0 & 0 & 0 & 0 & 0 & 0 & 0 & 0 \end{array}\right]$$

**Figure 3:**
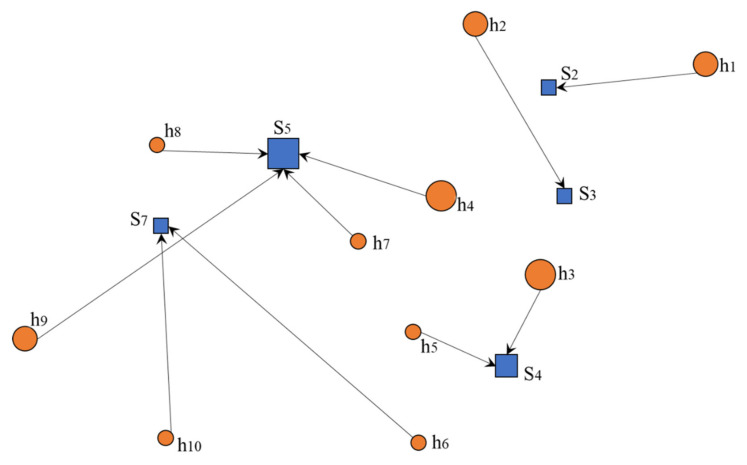
Schematic diagram of the L-A model results.

It can be seen that the evacuation routes of the evacuees are (Figure 3): residential area *j* = 1 is allocated to the shelter *k* = 2; residential area *j* = 2 is allocated to the shelter *k* = 3; residential area *j* = 3 is allocated to the shelter *k* = 4; residential area *j* = 4 is allocated to the shelter *k* = 5; residential area *j* = 5 is allocated to the shelter *k* = 4; residential area *j* = 6 is allocated to the shelter *k* = 7; residential area *j* = 7 is allocated to the shelter *k* = 5; residential area *j* = 8 is allocated to the shelter *k* = 5; residential area *j* = 9 is allocated to the shelter *k* = 5; and residential area *j* = 10 is allocated to the shelter *k* = 7.

④ The minimum total evacuation time for all evacuees is 74,000 min, and the number of evacuees is 9400. The average shelter evacuation time is 7.87 min/per, and all people’s evacuation time is within the maximum allowable time.

⑤ The total construction area of all shelters combined is 19,000 m^2^, which is a short-term resident shelter that can accommodate a total population of 9500. The total number of evacuees is 9400. According to the redundancy of the population, it can shelter 100 more people. Therefore, the use efficiency of the shelter is 9400÷9500=98.94%.

It can be seen that the L-A model of the shelters constructed in this study not only satisfies the constraints of cost, but also minimizes the evacuation time of all people in the area and maximizes the use efficiency of the shelters.

## 5. Conclusions

Based on the three core objectives of fairness, efficiency, and cost, this study constructs a multi-objective and bi-level L-A model to maximize the economic and social utility and determine the shelter locations and service areas. Solving the contradiction between economic governmental investment and fairness to evacuees improves the city’s ability to cope with extreme disasters. 

This study enriches the objectives of the multi-objective location model theory and puts forward an objective principle of maximizing the comprehensive utility. On the one hand, it emphasizes the economic utility of low investment and a high utilization rate of facilities. On the other hand, it considers the social utility that meets the demands and behaviors of evacuees. In the aspect of economic utility, the former location model has the single consideration of the economy of the shelter, and the only standard to measure the cost of investment is the number of shelters. In fact, from a long-term perspective, the economic sustainability of shelters should consider not only the construction investment but also the utilization efficiency of facilities and later operation and maintenance costs. This study explores the best scheme from the two perspectives of the construction number and scale. At the same time, the shelter economy is considered more comprehensively in the model design, and the use efficiency of the shelters is maximized as an important evaluation index. In terms of social benefits, this study not only continues to pay attention to the fairness and efficiency of shelters but also considers the influence of practical factors on the behavior of evacuees, such as the attractiveness of the distance and scale of the shelters to the evacuees and the capacity constraints of the shelter, which more closely relates to evacuees’ decisions under real conditions.

The bi-level multi-objective L-A model is improved in this study. The gravity model is introduced into the AEE location model, which expresses the preference of evacuees for the distance and scale of the shelter as a function, making the results more objective and realistic. According to the different key objectives and characteristics of different location stages, the multi-model method is used to integrate the SCLM and PMM, which can be used to solve the location problem with an uncertain number and spatial location of shelters. The simulated annealing method is used to solve the model, and the solution of the example proves the operability and high utility of the model in a practical application, which provides scientific support for the decision-makers and public. This model can be used in other countries and regions to aid in shelter site selection decisions.

The proposed model also has some limitations. The model uses the assumption from the gravity model that the behavior characteristics of evacuees are based on the premise that the evacuees make rational judgments, such as the priority of choosing large-scale and short-distance shelters. However, in actual emergency evacuations, human psychology is quite complex, and it is easy to blindly follow the crowd to make irrational choices, and there are individual differences. Therefore, research on evacuees’ requirements based on group psychology and behavioral characteristics in an emergency situation should be developed in future shelter plans and site selections. At the same time, it also reflects the importance of strengthening public emergency education and exercise in emergency management. This urgently requires the combination of emergency management agencies, urban planners, communities, organizations, and other forces to strengthen through policies, communication, and implementation strategies.

Nevertheless, the AEE model proposed a quantifiable strategy to optimize shelter locations, which can solve the main problems in the current planning of shelters and promote the further improvement and development of the bi-level multi-objective L-A model for shelters.

## Figures and Tables

**Table 1:** Classical location model of public facilities from the perspective of shelter location.

Model Type	Optimization Objectives	Characteristics	Advantages	Disadvantages
P-median model (PMM)	Minimize total weighted distance from the evacuation demand sites to the shelters	Number of facilities known; Find the most suitable location	Efficiency first; Consider fairness; Minimum cost with a known number of facilities	Neglect the influence of the shelter’s service capacity (e.g., level or scale)
P-center model (PCM)	Minimize the maximum distance from the evacuation demand sites to the shelters	Number of facilities known; Find the most suitable location	Fairness first; Minimum farthest evacuation distance	High cost; Easy to cause waste of resources; Neglect the preference of evacuees
Set covering location model (SCLM)	Minimize the number of shelters under the premise of evacuation demand sites full covered	Find the minimum number of facilities and the most suitable location	Consider fairness and achieve full coverage; Minimum number of facilities	Neglect the constraint of facility scale and the distribution of existing facilities
Maximal covering location model (MCLM)	Maximize the service capacity of shelters within the cost constrain	Number of facilities known; Make facilities cover the largest number of evacuation demand sites	Maximize coverage; Highest utilization of available shelters	Insufficient fairness; Unable to ensure full coverage of evacuation demand sites; Neglect the constraint of facility scale

**Table 2:** Literature review on the study of location and allocation of shelters.

Time	Authors	Objective Hierarchy	Main Model	Objectives	Constraint	Solution Methods	Objects
1971	Toregas et al. [29]	Single	SCLM	Minimum number	Distance	Linear programming	Emergency facility
1991	Sherali et al. [23]	Single	PMM	Minimum time	Capacity	Heuristic and an exact implicit enumeration algorithm	Hurricane shelter
1997	Adenso-Díaz & Rodríguez [34]	Single	MCLM	Maximum coverage	Distance Number	Tabu search metaheuristic	Ambulance bases
2001	Zhou & Jian [35]	Single	MCLM	Maximum coverage	Distance Number	Exact algorithm	Emergency shelter
2004	Huang et al. [25]	Single	PMM	Minimum distance	Number	Genetic algorithm	Earthquake shelter
2005	Kongsomsaksakul et al. [24]	Multi	Bi-level programming	Minimum cost Minimum time	Capacity	Genetic algorithm	Flood shelter
2005	Chen, Z.Z., & You, J.X. [51]	Multi	Hierarchical location (SCLM + MCLM)	Minimum number Maximum coverage	Distance Capacity	Exact algorithm	Ambulance center
2006	Li et al. [44]	Multi	MCDM(AHP)	Minimum risk (include 7 factors)	Distance Capacity	Weighted Voronoi diagram	Fixed shelter
2006	Zhou et al. [52]	Single	PMM + AHP	Minimum distance	Capacity	Approximation algorithm	Emergency shelter
2007	Li et al. [53]	Multi	Bi-level programming	Minimum cost Maximum capacity	Capacity	Iterative calculation	Emergency shelter
2008	Xu et al. [54]	Multi	Hierarchical location (SCLM + MCLM)	Minimum number Maximum coverage	Distance	GIS-based decision support system	Emergency shelter
2009	Pan [41]	Single	PMM	Minimum distance	Capacity	Genetic algorithm	Typhoon shelter
2009	Alcada-Almeida et al. [55]	Multi	Multi-PMM	Minimum distance Minimum risk Minimum time	Capacity Number	GIS-based decision support system	Fire shelter
2009	Saadatseresht et al. [56]	Multi	Spatial MOP	Minimum risk Minimum distance	Distance Capacity	NSGA-II and GIS	Safe area
2010	Wei [57]	Multi	MCLM	Maximum coverage	Distance Number	Exact algorithm	Emergency resources
2010	Chen et al. [58,59]	Multi	Hierarchical model	Minimum distance Minimum cost	Capacity	General optimizer (LINGO)	Emergency shelter
2010	Zhou et al. [60]	Multi	MCLM + PMM	Maximum coverage Minimum distance	Distance Nonoverlapping	General optimizer (LINGO)	Earthquake shelter
2010	Ng et al. [49]	Multi	Bi-level programming	Minimum cost Minimum time	Capacity	Simulated annealing algorithm	Emergency shelter
2011	Huang et al. [43]	Single	SCLM + Network analysis	Maximum coverage	Capacity Distance	GIS-based decision support system	Earthquake shelter
2011	Wu, J. & Weng, W. [61]	Multi	SCLM + Network analysis	Minimum cost Minimum number Minimum risk	Distance	GIS-based decision support system	Emergency shelter
2011	Li et al. [62]	Single	PMM	Minimum distance	Capacity Continuity	Shift insertion	Emergency shelter
2012	Coutinho-Rodrigues et al. [63]	Multi	Spatial MOP	Minimum distance Minimum risk Minimum time Minimum number	Capacity Number	GIS-based decision support system	Fire shelter
2012	Chu et al. [64]	Single	MCDM (AHP)	Maximum weight	Distance	Linear programming	Central refuge
2012	Liu [65]	Multi	Hierarchical location (SCLM + PMM, SCLM + MCLM)	Minimum number Maximum coverage	Distance Cost	GIS-based decision support system + Approximation algorithms	Earthquake shelter
2013	Ma [66]	Multi	SCLM + MCLM	Maximum coverage Minimum cost Minimum distance	Capacity Number	Lagrange method	Emergency shelter
2014	Liu and Zhong [46]	Multi	MCDM (AHP)	Maximum weight	Accessibility Capacity	Linear programming	Earthquake shelter
2014	Wang et al. [67]	Multi	MCDM (TOPSIS) + SCLM	Minimum cost Maximum coverage Minimum distance	Distance Number	Genetic algorithm particle swarm optimization	Earthquake shelter
2014	Chu [68]	Multi	MCLM + PMM + MCDM (TOPSIS)	Minimum number Minimum distance	Capacity Distance Nonoverlapping	GIS-based decision support system + Particle swarm optimization	Earthquake shelter
2014	Li et al. [69]	Multi	Spatial MOP	Minimum distance Maximum coverage Minimum number	Capacity Distance Nonoverlapping	GIS-based decision support system	Fixed shelter
2015	Kilci et al. [28]	Single	PCM	Maximum weight	Capacity	GIS-based decision support system	Temporary shelter
2015	Yuan et al. [70]	Single	SCLM	Maximum coverage	Capacity Number	Genetic algorithm	Fixed shelter
2015	Chu et al. [71]	Multi	MCLM + PMM	Minimum number Minimum distance	Capacity Nonoverlapping	General optimizer (LINGO)	Fixed shelter
2015	Ma et al. [72]	Multi	AHP + EVM + PCM	Maximum weight Minimum risk Minimum distance	Capacity Nonoverlapping Distance	Particle swarm optimization	Fixed shelter
2016	Xu et al. [73]	Multi	MCDM (AHP)	Maximum weight (Suitability, Feasibility, Sustainability)	-	Linear weighted sum	Flood shelter
2017	Chen [27]	Multi	Bi-level Programming (MCLM + PMM)	Minimum number Minimum distance	Capacity Distance Nonoverlapping	General optimizer (LINGO)	Fixed shelter
2017	Boonmee et al. [50]	Multi	Bi-level Programming	Minimum distance Minimum risk	Number Capacity Demand	Gurobi optimizer	Flood shelter

## Data Availability

Not applicable.
